# Usefulness of Preoperative Surgical Simulation with Three-Dimensional Fusion Images for Resection of Cerebral Cavernous Malformations Near Broca's Area

**DOI:** 10.1155/2014/853425

**Published:** 2014-04-14

**Authors:** Satoshi Takahashi, Yoshio Tanizaki, Kazunori Akaji, Tadashige Kano, Kenji Hiraga, Ban Mihara

**Affiliations:** ^1^Department of Neurosurgery, Institute of Brain and Blood Vessels, Mihara Memorial Hospital, Ohta-machi 366, Gunma, Isesaki 372-0006, Japan; ^2^Hiraga Neurosurgical Clinic, Gunma, Tamamura 370-1124, Japan; ^3^Department of Neurology, Institute of Brain and Blood Vessels, Mihara Memorial Hospital, Gunma, Isesaki 372-0006, Japan

## Abstract

Treating subcortical brain lesions in or near eloquent areas is challenging not only because lesions must be resected while preserving brain tissue involved in essential functions, but also because lesions often cannot be easily identified from the surface of the brain. Here, we report 2 cases of cerebral cavernous malformations near Broca's area. In both cases, lesions were surgically removed by utilizing three-dimensional fusion images created using preoperative magnetic resonance imaging and computed tomography data. Excisions were completed without any worsening of speech function, and the use of presurgical simulations was found to be useful in the design and execution of the actual operations. The technique described in this report serves as a useful tool in simulating surgical strategies by using brain gyri and sulci as surgical landmarks. Furthermore, in contrast to other intraoperative techniques, this method can aid in shortening the duration of surgery and can help limit damage to eloquent areas of the brain.

## 1. Introduction

Cerebral cavernous malformations are relatively benign lesions that frequently remain clinically undetectable [1]. Thus, asymptomatic cerebral cavernous malformations are generally treated conservatively, and only their symptomatic counterparts are considered for surgical interventions [2].

Cerebral cavernous malformations exist in the subcortical regions surrounded by the brain parenchyma, and it is sometimes difficult to determine their location from the surface of the brain during surgery. Although brain navigation is a powerful tool in identifying the location of the lesions, it is a time consuming method and can be affected by brain shifts during surgery [3].

Functional mapping is also very important at the time of surgical intervention, especially when the cerebral cavernous malformation is located near an eloquent area or deep inside the brain. For cerebral cavernous malformations near Broca's area, functional magnetic resonance imaging (fMRI), navigated transcranial magnetic stimulation (nTMS), or awake surgeries are the methods of choice. However, anatomical identification of the region (the inferior frontal gyrus) is also important. In this context, three-dimensional (3D) images can enable surgical teams to better understand the anatomy of the surgical field.

In this report, we introduce our surgical strategy to remove cerebral cavernous malformations near Broca's area. We first created 3D fusion images using data from magnetic resonance (MR) and computed tomography (CT) images. Using these fusion images we simulated surgical procedures on a commercially available workstation. We think that this method, in combination with preoperative functional mapping, can be used for minimally invasive removal of cerebral cavernous malformations near Broca's area. Our method does not require a navigation system and can also be used for surgeries of other brain lesions buried inside the brain parenchyma.

## 2. Case Presentation

### 2.1. Preparation of Preoperative 3D Fusion Images

Within a 1-month period, we surgically treated 2 patients with cerebral cavernous malformations located near Broca's area. In both patients, the cerebral cavernous malformations were symptomatic; thus, surgical removals were recommended.

In an attempt to remove the lesions with minimal invasiveness, we constructed and utilized preoperative 3D fusion images for surgical simulation. Surgical simulations were performed for the following purposes:

1. to identify a surgical route to the lesions, using sulci and gyri as landmarks,
2. to design a minimally invasive procedure for craniotomy and skin incision, and
3. to identify the anatomical location of Broca's area (using the inferior frontal gyrus as a marker).

MR imaging (GE Medical Systems) and CT scanning (Toshiba Medical systems) data sets were transferred to Ziostation (Ziosoft, Tokyo, Japan), a 3D workstation. Using the workstation, a 3D model of the brain surface and arteries was reconstructed from the MR images, and a 3D model of the skull, hematoma, and skin was reconstructed from CT scans. Use of Ziostation allows neurosurgeons to rotate or magnify 3D fusion images. Additionally, each element (the skull, brain, or arteries) can be dissected on the workstation so that a neurosurgeon can preoperatively simulate surgical procedures.

### 2.2. Case 1

#### 2.2.1. Onset

**Figure 1:**
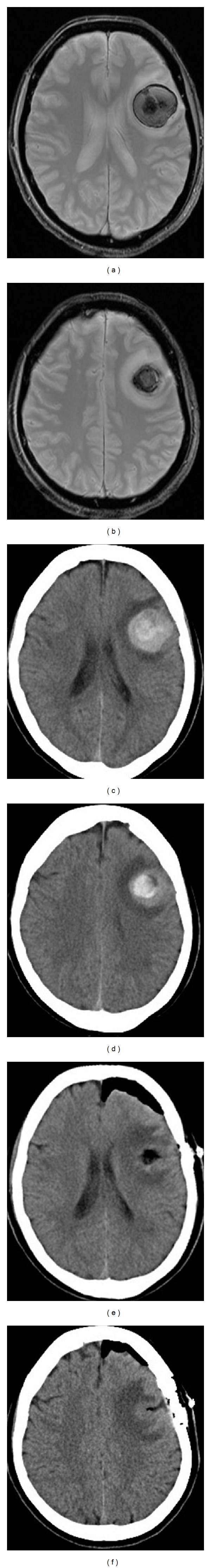
MR images and CT scans of Case 1. (a) and (b), preoperative MR images; (c) and (d), preoperative CT scans; (e) and (f), postoperative CT scans.

A 52-year-old right-handed woman with no prior history of neurological symptoms was referred to our hospital with chief complaints of aphasia and headaches and no sign of both extremities weakness. These symptoms had developed suddenly 1 week before she was admitted to the hospital. CT images revealed intracerebral haemorrhage (maximum 3.5 cm in diameter) in the left frontal lobe (Figures 1(c) and 1(d)).

#### 2.2.2. Initial Treatment

**Figure 2:**
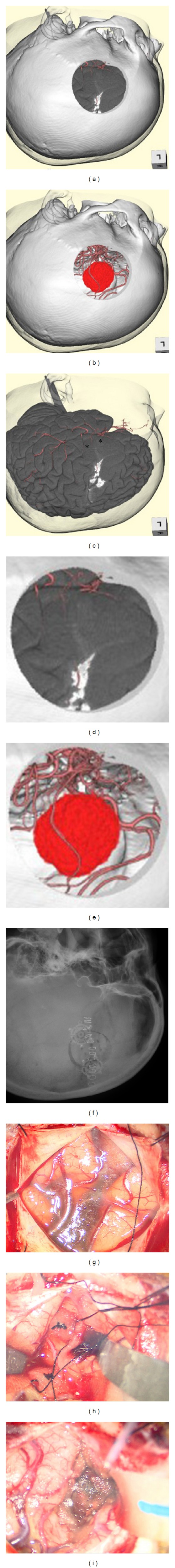
Case 1. (a)–(e), 3D fusion images on the workstation; (c), anatomical estimation of Broca's area (∗∗) located caudal to the lesion; (f), plain craniogram obtained after surgery; (g)–(i), intraoperative images; (g), image taken just after opening the dura to identify the preoperatively planned sulci and gyri; (h), image taken at the time of corticotomy; (i), image taken after haemostasis was accomplished.

Cerebral angiography revealed a contralateral unruptured aneurysm in the M1 region (not shown). On the basis of the preoperative MRI findings (Figures 1(a) and 1(b)), the patient was diagnosed with intracerebral haemorrhage (ICH) caused by cerebral cavernous malformation. We recommended surgical intervention since the lesion was symptomatic and we thought that there would be a future risk of rebleeding. A preoperative 3D fusion image was then constructed to simulate surgical procedures (Figures 2(a)–2(e)). From the results of the preoperative simulation, we determined that a straight skin incision and small craniotomy behind the hairline would be enough to remove the hematoma (Figure 2(f)). Using the simulated 3D image, Broca's area (the inferior frontal gyrus) was identified to be caudal to the hematoma cavity (Figure 2(c)). This information led the surgical team to avoid contact with the caudal wall of the hematoma cavity so that Broca's area was not damaged.

#### 2.2.3. Craniotomy

The patient underwent craniotomy under general anaesthesia 1 week after the onset of haemorrhage. A straight skin incision was made behind the hairline, and a bone flap was removed to reveal the preoperatively targeted sulcus (Figure 2(a)). A cross-shaped opening was made in the dura (Figure 2(g)) followed by a large opening in the arachnoid membrane over and inside the targeted sulcus (Figure 2(h)). Inside the sulcus, a gyrus flattened by the hematoma was observed as indicated in the preoperative simulation. A minimal corticotomy (4 mm) was then made on the gyrus to reach the hematoma (Figure 2(h)). Most of the hematoma was liquid and was easily removed by suction; however, detachment of the solid part from the surrounding brain was difficult. Haemostasis was accomplished after removing the solid portion of the lesion (Figure 2(i)) that was pathologically identified as the cerebral cavernous malformation.

#### 2.2.4. Postoperative Course

A CT scan obtained after the surgery confirmed complete removal of the hematoma (Figures 1(e) and 1(f)). The preoperative symptom of motor aphasia improved immediately after the operation. The patient was discharged from hospital after 1 month following speech rehabilitation. At the time of discharge, motor aphasia of the patient was almost alleviated.

### 2.3. Case 2

#### 2.3.1. Onset

**Figure 3:**
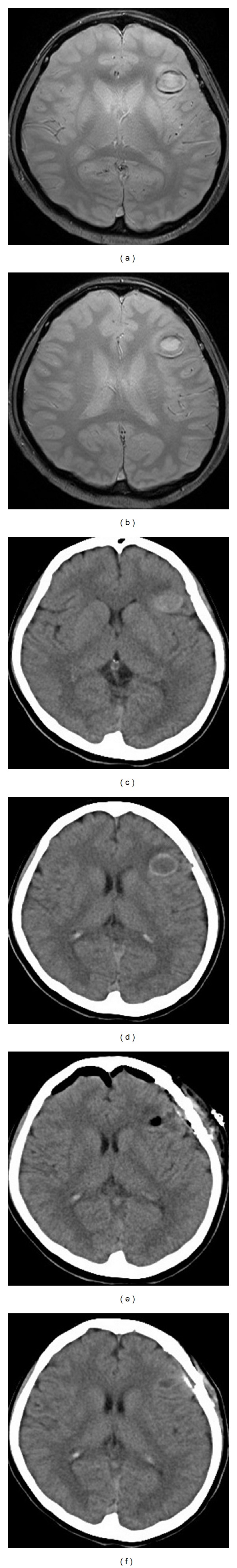
MR images and CT scans of Case 2. (a) and (b), preoperative MR images; (c) and (d), preoperative CT scans; (e) and (f), postoperative CT scans.

A 23-year-old right-handed woman had experienced refractory ICH in the left frontal lobe 3 times within 2 years and was referred to our hospital. A CT scan revealed a small high-density region in the left frontal lobe that was confirmed to be a subacute ICH (maximum 2.0 cm in diameter) (Figures 3(c) and 3(d)). On admission, the patient was alert and oriented. The patient did not show any neurological abnormalities other than headache.

#### 2.3.2. Initial Treatment

**Figure 4:**
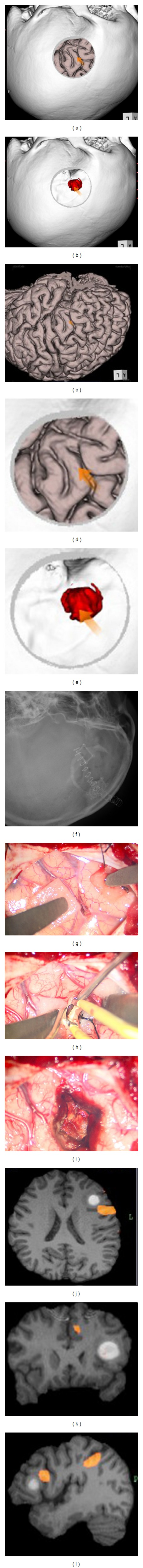
Case 2. (a)–(e), 3D brain images on the workstation; (g)–(i), intraoperative pictures; (j)–(l), results of fMRI. The speech centre (orange regions) was located posterior to the lesion; (f), plain craniogram obtained after surgery; (g), image taken just after opening the dura to identify the preoperatively planned sulci and gyri; (h), image taken at the time of corticotomy; (i), image taken after haemostasis was accomplished.

A cerebral angiography revealed no abnormalities. On the basis of preoperative MRI findings (Figures 3(a) and 3(b)) as well as the clinical course of the patient, a preoperative diagnosis of refractory ICH caused by cerebral cavernous malformation was made. Our recommendation to the patient was surgical removal of the lesion because it bled frequently and was growing in size. A preoperative 3D fusion image was then acquired to simulate the surgical procedure (Figures 4(a)–4(e)). From the result of the simulation, we determined that a straight skin incision and small craniotomy would be enough to remove the hematoma (Figure 4(f)). To identify Broca's area, a preoperative fMRI was performed and Broca's area was estimated to be positioned posterior to the hematoma (Figures 4(j)–4(l)). This information led the surgical team to avoid touching the posterior wall of the hematoma cavity so that Broca's area was not affected.

#### 2.3.3. Craniotomy

Following surgical simulation, the patient underwent a craniotomy under general anaesthesia. A curvilinear incision was made, and it ended just behind the hairline. A bone flap was then removed to reveal the preoperatively targeted sulcus. A cross-shaped opening was made in the dura (Figure 4(g)) followed by a large opening in the arachnoid membrane over and inside the targeted sulcus. From the results of the preoperative simulation on the workstation, we determined that the hematoma of the patient would be located underneath the bottom of the targeted sulcus (Figure 4(h)). Thus, a minimal corticotomy (4 mm) was made on the bottom of the sulcus where an old hematoma was discovered and was carefully detached from the surrounding brain. Haemostasis was accomplished after removing the solid portion of the hematoma (Figure 4(i)), which was pathologically identified as a cerebral cavernous malformation.

#### 2.3.4. Postoperative Course

A CT scan obtained after surgery confirmed complete removal of the hematoma (Figures 3(e) and 3(f)). The preoperative symptom of headache was relieved immediately after the operation, and the patient was discharged from hospital 1 week after surgery without any neurological sequel.

## 3. Discussion

The management of cerebral cavernous malformations typically includes 2 options: surgery or conservative observation. One of the most important aspects in treating these lesions is the appropriate selection of patients for surgery because most of these intracranial malformations are benign [1]. In cases where intervention is recommended, surgery is most suitable for patients with symptomatic cerebral cavernous malformations and usually becomes the most advisable option in cases with seizure and/or haemorrhage [2]. Surgical strategy is relatively straightforward in patients with cerebral cavernous malformations in noneloquent supratentorial areas. However, intervention becomes more complex in patients with symptomatic cerebral cavernous malformations near eloquent areas or in deep brain regions [1]. In the first case, we recommended surgery because it was a symptomatic lesion and we hoped that removal of hematoma could relieve the patient's symptom of motor aphasia. In the second case, we recommended surgery because of recurring frequent bleeding (at least 3 times in a 2-year period) as well as growth of the lesion. In both cases, the option of conservative observation was fully explained since lesions in each of the cases were located near Broca's area. In the end, the final decision to undergo surgery was made by the patients themselves.

Functional mapping and/or neuronavigation have proven to be useful tools during surgery in patients affected by symptomatic cerebral cavernous malformations located near eloquent areas [2, 4]. In the past, intraoperative direct electrical stimulation (DES) of brain tissue was the only modality available for brain mapping [5]. In recent decades, a considerable amount of effort has been made on developing various technologies for noninvasive preoperative brain mapping. Advancement in this field would assist many surgical situations, including the charting of verbal function by using DES since patients undergoing this surgery are required to remain awake. Awake surgeries are often difficult for patients and can only be undertaken by experienced institutions with skilled anaesthesiologists. One reasonable method for identifying the location of the verbal cortex with minimal invasiveness is through using fMRI. This technique has been used for surgeries in patients with supratentorial cerebral cavernous malformations in eloquent brain areas [6]. In Case 2, we performed fMRI to identify the location of Broca's area. This information enabled the surgical team to avoid the posterior wall of the hematoma cavity in an effort to protect Broca's area.

A relatively new method for functional brain mapping, nTMS, has gained popularity in the field of brain mapping [5]. Only TMS is analogous to the “gold standard” of DES because it allows for electrical stimulation of the brain and observation of induced effects. This technique has successfully been used in the preoperative identification of Broca's area [7], and if it becomes more widely available, nTMS will serve as a powerful tool for cerebral cavernous malformation surgery in eloquent areas. When using neuronavigation, shifts in the brain after dura opening are common and should be accounted for [3]. Intraoperative MRI may overcome the problem of brain shifts; however, it is still a time-consuming method [8].

Our concept of preoperative surgical simulation using a brain 3D fusion image partly resembles MRI-based corticotopography (MRI-bct) as described by Esposito et al. [3, 9]. Both concepts use anatomical landmarks such as the sulci, gyri, and cortical vessels to determine lesion location [9]. The main advantages of our method over MRI-bct are as follows: (1) our method can provide information on the skull and face (ear, nose, etc.) and can aid in designing the skin incision and craniotomy, and (2) our method provides the option of gradual simulation of the surgical procedures by using the “cut” function of the workstation.

In the present cases, we adopted a transsulcal microsurgical approach [10, 11] to minimize brain tissue injury. Approaching the subcortical lesions by opening the sulci enabled us not only to shorten the distance between the surface of the brain and the lesions, but also to utilize the sulci themselves as anatomical landmarks.

Here, we report 2 cases of patients with cerebral cavernous malformations near Broca's area. With the aid of presurgical simulation using 3D fusion images, lesions in both the cases were surgically removed without neurological sequel. The main advantages of our technique are that it offers the ability to simulate surgical strategy and to utilize brain gyri and sulci as surgical landmarks. Furthermore, in contrast to other intraoperative techniques, this method can aid in shortening the duration of surgery.
